# Trans oral endoscopic surgery (TOES) of para-pharyngeal space tumours: single surgeon experience

**DOI:** 10.1007/s00405-025-09600-9

**Published:** 2025-11-17

**Authors:** Hesham S. Kaddour, Amir Habeeb, Mohamed Amin, Mohamed S. Rashwan

**Affiliations:** 1 Department of Otolaryngology, Barking, Havering and Redbridge University Hospitals NHS Trust, Rom Valley Way, RM7 0AG Romford, Essex England; 2 Department of Otolaryngology, Head and Neck Surgery, Faculty of Medicine, Suez Canal University, Ismailia, Egypt; 3 Present Address: Queen Mary University of London, Mile End Road, London, E1 4NS England

**Keywords:** Otorhinolaryngology, Head and neck surgery, Outcomes, Pathology

## Abstract

**Purpose:**

Parapharyngeal (PP) tumours are rare and form 0.5% of head and neck pathology. The anatomy of PPS is divided into pre-styloid space and post-styloid which contains the carotid sheath, lower four cranial nerves and the sympathetic chain. The pathology of the PP space is heterogenous, but the majority are benign mainly from the deep lobe of the parotid. The purpose of our study was to map our experience of performing transoral endoscopic surgery (TOES) for removing these tumours and highlight some potential benefits over traditional transcervical approaches.

**Methods:**

Retrospective study looking at 9 patients operated on by a single head and neck surgeon for benign pre-operative investigations after discussion in a multidisciplinary team setting. Data was collected such as gender distribution, size and laterality of tumour, post operative histology and whether there was recurrence or incomplete excision.

**Results:**

9 patients ( Male = 6, Female = 3, Age 47) were operated on via TOES. Their average tumour sizes were 46 × 36 mm with a right sided predominance *N* = 7. Post operatively 78% (*N* = 7) were benign in histology with 11% ( *N* = 1) requiring revision surgery for recurrence and 11% (*N* = 1) having incomplete excision.

**Conclusion:**

TOES is advantageous as it is minimally invasive surgery (MIS), scar less, associated with quicker recovery, shorter length of stay (LOS) and subsequently is cost effective. However, it has also its drawbacks like being suitable in only selective cases, limited approach, the need for special instruments, being an unclean procedure and finally its learning curve.

**Supplementary Information:**

The online version contains supplementary material available at 10.1007/s00405-025-09600-9.

## Introduction

The parapharyngeal space (PPS) is an inverted pyramidal potential fascial space extending from the skull base down to the hyoid bone level in the neck. Additionally, the PPS is bound anteromedially by the buccopharyngeal fascia that surrounds the superior constrictor muscle as well as laterally by the medial pterygoid muscle and the prevertebral fascia posteriorly. The PPS is divided by the styloid process into a pre and post-styloid compartments [1]. 

Tumours that arise in PPS are rare and represent 0.5–1% of the head and neck tumours [1]. These lesions are heterogenous in types with broadly 80% being benign and 20% are malignant [2]. Patients may be asymptomatic or present with a bulging tonsil/soft palate, lateral neck lump, earache and/or lower cranial nerve palsy (IX – XII) depending on the size and the nature of the tumour [2]. 

When diagnosing PPS tumours, the symptoms and signs on presentation are critical but, more importantly, 3D imaging of the head and neck area such as with MRI, CT or angiograms allows visualisation and early diagnosis to facilitate further planning. Ultrasound fine needle aspiration cytology (USFNAC) should be considered as an option except in the context of vascular tumours. The management of PPS tumours is quite challenging due to the awkward position and their heterogenous nature. Traditionally, these tumours are approached surgically via external cervical incision with or without mandibulotomy depending on its nature, size and extension [2–4]. However, with the modern advanced technology natural orifice trans-luminal endoscopic surgery (NOTES) has a become safe, efficient and cost-effective alternative. We present our experience in managing PPS tumours via trans oral endoscopic surgery (TOES) as an alternative to the traditional external cervical approach [5–7].

## Patients and method

All patients with PPS tumours managed by the senior author (HSK) at the Otolaryngology, Head and Neck surgery department of Barking, Havering and Redbridge University Hospitals NHS Trust, United Kingdom during the period from January 2009 to December 2018 were reviewed. 9 out of 22 patients with PPS tumours were managed via TOES approach are included in this study. Inclusion criteria are patients with pre-styloid benign non-vascular PPS pathology. Exclusion criteria are patients with multiple co-morbidities and unfit for general anaesthesia, post-styloid tumours, vascular pathology who required external approach. Informed consent was obtained from all the patients for the surgical procedure and participation in the study was in accordance with the ethical standards and approval of the hospital ethical review board. 

**Fig. 1:**
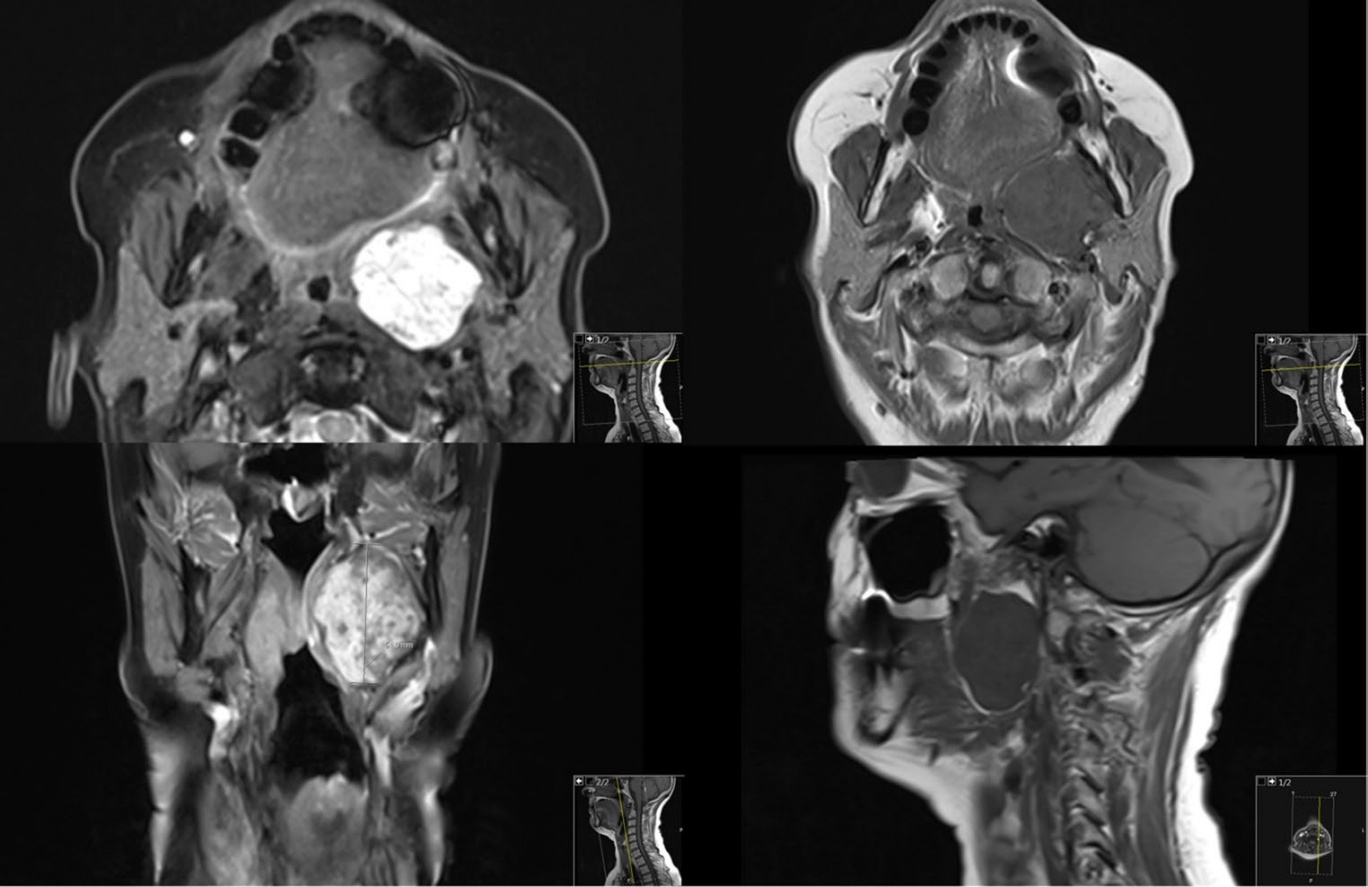
MRI Neck with contrast (axial, coronal and sagittal views with different weightings)

Pre-operative imaging including contrast enhanced magnetic resonance imaging (MRI) or computed tomography (CT) was used to establish the diagnosis, site, size and nature of the tumour. Computer tomography-angiography (CTA) or magnetic resonance-angiography (MRA) for vascular tumours such as carotid body tumours (CBT) was performed. Ultrasound guided fine needle aspiration cytology (USFNAC) was used to establish pre-operative histological diagnosis except for vascular lesions. All cases were discussed at the head and neck multidisciplinary team meeting (MDTM). Informed written consent was obtained from all the patients for the surgical procedure. Figure 1 shows a typical MRI neck with contrast for a pre-styloid benign parapharyngeal tumour.

All the procedures were performed under general anaesthesia using nasotracheal intubation. A Boyle-Davis mouth gag was used with the surgery performed by the senior author with an assistant holding the endoscope supported over the upper flange of the mouth gag whilst the main surgeon performed the surgery with high-definition video assistance. An incision was made extending from the soft palate down towards the lower pole of the tonsil with monopolar cautery. The mucosa, submucosa, and the superior constrictor muscle were divided and 2/0 Vicryl stay sutures to retract both anterior and posterior tonsillar pillars to gain access to the parapharyngeal space and expose the tumour. Extracapsular sharp and blunt dissection of the tumour was carried out using 0 and 45-degree 4 mm endoscopes (Storz), to help mainly visualising the superior and lateral aspects of the tumour with great care given to avoid any major vascular injury. Bipolar diathermy was used to cauterise small and medium sized vessels. The tumours were handled carefully to avoid rupture and removed intact for histological examination. The tumour bed was then washed with saline and haemostasis was achieved. The wound was closed in layers with 3/0 Vicryl. The below video illustrates the key operative steps described above.

## (VIDEO)

Post-operative care included prophylactic intra-operative intravenous (IV) antibiotic, IV dexamethasone, nasogastric tube (NG) feeding for 24 h. High dependency unit (HDU) admission was based on the patient’s condition and anaesthetic advice. All the patients were discharged home 24–48 h post-operatively with adequate analgesia and a patient leaflet advice for tonsillectomy.

All patients were followed up 2 weeks post-operatively when histopathological reports provided the final definitive diagnosis of the lesion and its complete or incomplete excision. Post-operative follow-up control MRI scan with contrast was performed 6–8 weeks later to check for any residual or tumour recurrence.

## Results

**Fig. 2:**
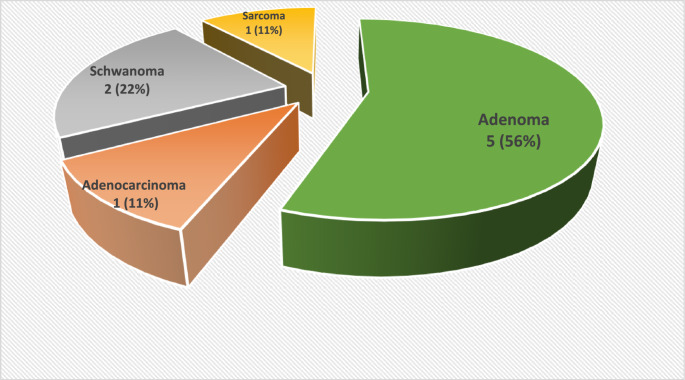
TOES Pathology (*N* = 9)

Summary of the cases are in Table 1; Fig. 2 below.

**Table 1:** Table showing the patients captured in the study, their demographics including age and gender alongside the size, laterality, pathology and excision status of the lesion

	AGE (years) Mean = 47	GENDER Male, M = 6 Female, F = 3	SIZE (mm) Mean = 46x36	SIDE Right, R = 7 Left, L = 2	PATHOLOGY	EXCISION
1	85	F	41x29	R	Neuroma	Complete
2	41	M	39x33	R	Pleomorphic Adenoma	Complete
3	17	M	34x31	R	Synovial Sarcoma	Complete
4	71	M	53x40	R	Pleomorphic Adenocarcinoma	Incomplete
5	38	M	42x30	L	Pleomorphic Adenoma	Complete
6	63	F	52x43	R	Pleomorphic Adenoma	Complete
7	35	M	60x43	R	Pleomorphic Adenoma	Complete Recurrence
8	52	M	51x42	L	Pleomorphic Adenoma	Complete
9	25	F	40x30	R	Schwannoma	Complete

## Discussion

The parapharyngeal space is a deep potential neck space shaped as an inverted pyramid extending from the base of the skull to the hyoid bone. It may be divided into two compartments on the basis of its relationship to the styloid process. The contents of the pre-styloid compartment include the minor or ectopic salivary gland, branches of the mandibular division of the trigeminal nerve, internal maxillary artery, ascending pharyngeal artery, and pharyngeal venous plexus, whereas those of the post-styloid compartment include the internal carotid artery, internal jugular vein, cranial nerves IX-XII, cervical sympathetic chain, and glomus bodies. Tumours arising in the PPS are rare and represent 0.5–1% of the head and neck tumours [1]. These lesions are heterogenous in nature, however 80% are benign and only 20% are malignant [2]. Our 9 cases of PPS tumours were similar to those reported in the literature with 22% malignant and 78% benign.

 Patients with PPS tumours may be asymptomatic until the tumour reaches up to 3 cm in size when they present with a variety of symptoms including neck lump (25%) or oropharyngeal swelling (20%), and dysphagia (20%) [3, 4]. Other less frequent symptoms may be dysphonia, dyspnoea, neck pain or rigidity, trismus, otalgia or cranial nerve palsy [5–7]. Cranial nerve palsy, neck rigidity and trismus are more common among malignant PPS tumours [8–10]. All our patients presented with oropharyngeal swelling, asymmetrical tonsils, globus sensation and/or neck swelling. One young patient presented with recent onset of heavy snoring. None presented with lower cranial never palsy or trismus suggestive of malignancy.

Imaging is essential in the diagnosis of these tumours. MRI is usually more sensitive and specific compared with CT as MRI provides more detailed information about the characteristics of the lesions in addition to the exact location and extension as well as whether there are any malignant features [3]. The parapharyngeal fat as well as the position of the carotid artery in relation to the tumour is very important to differentiate pre and post-styloid lesions and hence crucial for surgical approach planning [4, 7, 8]. However, CTA or MRA should be done if vascular pathology is suspected such as a carotid body tumour (CBT). Pre-operative cytology using USFNAC or core biopsy should be considered except in vascular tumours [4]. 

Management of PPS tumours is complex due to their anatomical position and possibility of multiple tumour types. We believe that these rare cases should be discussed at the head and neck multidisciplinary team meeting (MDTM) including the relevant clinicians such as head and neck surgeons, radiologists and pathologists to agree on a unified management plan. The majority of the PPS tumours are managed surgically. Traditionally, these tumours are approached surgically via external cervical incision with or without mandibulotomy depending on its nature, size and extension [3, 4]. Unfortunately, these external approaches have been associated with several post-operative complications; the most common was injury to the marginal mandibular nerve especially with the transcervical approach [6]. Other serious complications included injury to any of the last four cranial nerves and facial nerve injury during the trans-parotid approach. In addition to the above the external approach is associated with large scars and longer hospital stays incurring more financial costs for the hospital. However, with the modern advanced technology in the era of minimal invasive surgery (MIS), natural orifice trans-luminal endoscopic surgery (NOTES) like trans oral robotic surgery (TORS) or trans oral endoscopic surgery (TOES) have become safe, efficient, scarless and accompanied with a shorter length of stay in hospital alternatives [3]. TOES is more cost-effective alternative to TORS which is not available in most of ENT units worldwide.

There have been few published papers on the removal of PPS tumours via TOES which are retrospective study including few cases like our current study [3]. We acknowledge that our current study has its own inherent limitations of being retrospective of a few numbers of cases. We think this is because of the rarity and heterogenicity of these tumours. However, our study has the advantage of being done by only one surgeon which reflects its consistency. Another obstacle is the fact our study represents a single arm looking solely at TOES and does not do a direct comparison with external approach for example but relies on our experience.

Preoperative selection of cases suitable for TOES is very critical and we believe this approach should be considered only for non-vascular benign tumours of 50 mm or less in diameter in the pre-styloid compartment of the PPS. Important factors to consider include degree of trismus that the patient has which would ultimately limit the amount of intraoral exposure and proper equipment required if conducting TORS but not so much for TOES. The relationship to the carotid artery is crucial in surgical planning and tumours that displace the carotid laterally with a well defined plane are usually more amenable to TOES [5]. The superior and lateral extent should be studied pre-operatively as extension towards the skull base would lead to difficulties visualising intraoperatively and a higher risk of tumour capsule spillage and subsequent recurrence; Boyce et al. suggested lesions at least 10 mm from the skull base on coronal cross-sectional imaging should be amenable to TORS [10]. Li et al. have more recently published their experience in resecting 16 post-styloid tumours [3]. However, they had encountered some intraoperative issues warranting conversion to an open approach. All our 9 cases were done via TOES and none were converted into open approach during the procedure.

TOES like any other surgical technique however, has its own drawbacks which include its limited access but the use of wide angle scopes can aid in overcoming this hurdle preventing the need for conversion to an open approach. Another limiting factor is that you enter the PPS in the neck (clean site) through the oropharynx (unclean site), therefore prophylactic IV intra-operative were given to all the patients and we did not come across any post-operative infections. Post-operative pain and swallowing is another side effect so in our first 3 cases, NG feeding tubes were inserted with adequate analgesia. The rest of the cases were managed without NG tubes having usual post-operative protocols akin to a routine tonsillectomy.

The other major advantage of TOES over TORS is that almost all the surgical instruments and facilities are available in any modern head and neck surgery operating room. It can allow high definition visualisation of small vessels in the deeper part of the dissection to allow for better haemostasis when compared with external approach. Another positive is the ability to utilise the endoscope as a teaching/educational tool for surgical trainees intraoperatively but also to reflect on surgical technique when watching the video postoperatively. It can be showed the patient who may be curious about the dissection. Also TORS dissection lacks the bimanual palpation and associated tactile feedback that you can potentially get if you are doing TORS/transcervical approach. TORS does however offer 3 dimensional optical magnification in addition to multiple different viewing angles of the tumours and a freedom of movement provided by the robot arms that may not be achievable with TOES although there is a steep learning curve for a tool that is not common to head and neck surgery. However, TOES should be done by an experienced surgeon who can select the appropriate cases to perform safely.

## Conclusion

PPS tumours are rare, heterogenous but the majority are benign. The complexities of the anatomy have driven the need for a standardised approach to removing tumours surgically. Their diagnosis depends mainly on pre-op MRI/CT/angiogram and USFNAC. Managing these tumours is very challenging therefore a head and neck MDTM discussion should be considered. Surgery is the main treatment with our preferred approach being external or NOTES. TOES for *selective* cases is safe, effective and a cheaper alternative to TORS. Long term outcome data as well as larger study sample sizes will be crucial in observing whether it is associated with differing recurrence rates of tumours as well improved quality of life questionnaire scores when compared to traditional transcervical approaches.

## Supplementary Information

Below is the link to the electronic supplementary material.

(MP4 193 MB)
